# Preformulation and stability in biological fluids of the retrocyclin RC-101, a potential anti-HIV topical microbicide

**DOI:** 10.1186/1742-6405-8-27

**Published:** 2011-07-29

**Authors:** Alexandra B Sassi, Katherine E Bunge, Brian L Hood, Thomas P Conrads, Alexander M Cole, Phalguni Gupta, Lisa C Rohan

**Affiliations:** 1 Magee-Womens Research Institute, 204 Craft Avenue, Pittsburgh, PA, 15213, USA; 2 Department of Pharmaceutical Sciences, School of Pharmacy, University of Pittsburgh, 1104 Salk Hall, 3501 Terrace St., Pittsburgh, PA, 15261, USA; 3 Department of Obstetrics, Gynecology and Reproductive Sciences, Magee-Womens Hospital, 300 Halket St., Pittsburgh, PA, 15213, USA; 4 Department of Pharmacology & Chemical Biology and the Clinical Proteomics Facility, University of Pittsburgh Cancer Institute, University of Pittsburgh School of Medicine, 5150 Centre Avenue, Pittsburgh, PA, 15232, USA; 5 Department of Molecular Biology & Microbiology, Burnett School of Biomedical Sciences, University of Central Florida College of Medicine, 4000 Central Florida Blvd, Bldg 20, Room 236, Orlando, FL, 32816, USA; 6 Department of Infectious Disease and Microbiology, School of Public Health, University of Pittsburgh, Address, Pittsburgh, PA, USA

## Abstract

**Background:**

RC-101, a cationic peptide retrocyclin analog, has *in vitro *activity against HIV-1. Peptide drugs are commonly prone to conformational changes, oxidation and hydrolysis when exposed to excipients in a formulation or biological fluids in the body, this can affect product efficacy. We aimed to investigate RC-101 stability under several conditions including the presence of human vaginal fluids (HVF), enabling the efficient design of a safe and effective microbicide product. Stability studies (temperature, pH, and oxidation) were performed by HPLC, Circular Dichroism, and Mass Spectrometry (LC-MS/MS). Additionally, the effect of HVF on formulated RC-101 was evaluated with fluids collected from healthy volunteers, or from subjects with bacterial vaginosis (BV). RC-101 was monitored by LC-MS/MS for up to 72 h.

**Results:**

RC-101 was stable at pH 3, 4, and 7, at 25 and 37°C. High concentrations of hydrogen peroxide resulted in less than 10% RC-101 reduction over 24 h. RC-101 was detected 48 h after incubation with normal HVF; however, not following incubation with HVF from BV subjects.

**Conclusions:**

Our results emphasize the importance of preformulation evaluations and highlight the impact of HVF on microbicide product stability and efficacy. RC-101 was stable in normal HVF for at least 48 h, indicating that it is a promising candidate for microbicide product development. However, RC-101 stability appears compromised in individuals with BV, requiring more advanced formulation strategies for stabilization in this environment.

## Background

Microbicides are being investigated as a potential alternative for the prevention of HIV. Microbicide products would be applied vaginally or in the rectum before intercourse to prevent transmission and acquisition of sexually transmitted infections (STIs), mainly human immunodeficiency virus (HIV) [1,2]. Several microbicide candidates with different mechanisms of action are being investigated [3]. The interaction of microbicide drug candidates with human vaginal fluids can result in chemical modification of the drug by oxidation, hydrolysis, or proteolysis, thereby decreasing its potential for biological activity.

Defensins are cysteine-rich, cationic antimicrobial peptides expressed by the leucocytes and epithelial cells of mammals. Defensins have been shown to protect cells from *in vitro *infection by human immunodeficiency virus (HIV-1). Retrocyclins (θ-Defensin) are the evolutionary descendants of α-defensin genes. Retrocyclins are circular 18-residue, tetracyclic peptides with three cysteine disulfide bonds. RC-101 (GICRCICGKGICRCICGR), a cationic retrocyclin analog synthesized by solid phase peptide synthesis, has shown activity against X4 and R5 strains of HIV-1 *in vitro *[4]. The mechanism occurs by preventing six-helix bundle formation of gp41 (a 41,000 MW glycoprotein), conferring a strong mechanism of protection against HIV-1 [5]. As a result, RC-101 has been identified as a potential microbicide candidate to prevent mucosal transmission of HIV-1 [5].

Biopharmaceuticals (proteins and peptides) have demonstrated advantages over small molecule microbicides. Biopharmaceuticals are more specific to the target, offer less adverse effects, and provide a more effective treatment. However, it is challenging to formulate a protein or peptide into a microbicide product. The product must overcome *in vivo *barriers that will affect efficacy of the product. Changes in efficacy can be related to: 1) protein modification, mostly due to conformational changes; 2) chemical degradation in the drug delivery vehicle; 3) proteolytic inactivation in the vaginal lumen, and/or 4) low penetration of the drug into the mucosal tissue [6]. It is crucial to understand, through a complete pre-formulation study, how conditions of temperature, pH, and oxidative effects will affect the protein or peptide. A preformulation study will expedite formulation of a successful microbicide product.

Vaginal fluid covers the vaginal epithelium and protects against entry of pathogens into deeper tissues. Cervical mucus has similar functions and additionally facilitates sperm penetration by changing its viscoelastic properties during ovulation. Properties of the mucus layer can either facilitate or impede the efficacy of a drug product. When a vaginal microbicide product is applied, its presence should not disrupt the natural protective mechanisms associated with the mucus layer. In some cases, vaginal fluids may be disadvantageous. The presence of physiological fluids may alter the characteristics of a vaginal product, which can reduce the overall efficacy of the drug substance, increase leakage, and decrease drug residence time at the target tissue [7]. More importantly, enzymatic activity and the presence of hydrogen peroxide produced by Lactobacillus greatly affect the stability of protein and peptide microbicide agents. This enzymatic barrier in vaginal fluid has been identified as a major barrier to the delivery and absorption of microbicides and other drugs [8].

The purpose of this study was to determine the stability of RC-101 in several conditions including the presence of human vaginal fluids, to describe the degradation pathways, and to investigate the protective effects of excipients against oxidation. In this study, several pre-formulation evaluations were performed for RC-101 to provide information needed to develop vaginal formulations of RC-101 for use as a topical microbicide product. This characterization included an evaluation of the stability of RC-101 in the presence of vaginal fluids, selected conditions of temperature, pH, and the presence of hydrogen peroxide.

## Methods

### Materials

Retrocyclin-1 (RC-101) was synthesized by the Peptide Synthesis Facility at the University of Pittsburgh (Pittsburgh, PA). As part of quality control of the material, mass spectrometry using a Quattro II triple quadrupole mass spectrometer electrospray ionization (Fisons Inc., Valencia, CA) and AU-PAGE were conducted to confirm identity and the molecular weight of the compound, and *in vitro *activity using TZM cells was conducted to confirm bioactivity of RC-101 against HIV-1. Acetonitrile (HPLC grade), trifluoracetic acid (TFA), sodium phosphate dibasic, phosphoric acid (85%), sodium acetate, and glacial acetic acid were obtained from Fisher Scientific (Fair Lawn, NJ). Urea was purchased from Spectrum Laboratory Products Inc. (Gardena, CA). Polyvinyl alcohol (PVA) was obtained from Kuraray America Inc. (New York, NY). Glycerin was obtained from Dow Chemical Company (Midland, MI). All other materials were obtained from Sigma (St. Louis, MO). De-ionized water was prepared from house-distilled water with a Milli Q (Millipore, Milford, MA) water system operating at 18.2 MΩcm.

### Pre-formulation studies

For all pre-formulation studies described below, triplicate solutions of RC-101 (500 μg/mL) were prepared in either water or aqueous buffer solution. Thermal degradation studies were conducted at 25, 37, and 65°C for a minimum period of 1 week. The effect of pH on the stability of RC-101 was evaluated over the pH range from 3 to 12 using 10 mM phosphate buffer solutions, at low (50 mmol/kg) and high (500 mmol/kg) ionic strength. Oxidation of RC-101 was evaluated by exposing a solution of RC-101 to hydrogen peroxide (H_2_O_2_) at concentrations of 3.0, 0.08, and 0.002% (v/v). The high H_2_O_2 _concentration (3.0%) was selected as a forced degradation concentration. More biologically relevant concentrations (0.002% and 0.08% H_2_O_2_) were selected based on reported studies which determined the amount of hydrogen peroxide produced by Lactobacillus present in the normal vaginal flora, and estimated calculations based on concentrations of Lactobacillus present [9,10]. Protection against oxidation was investigated by the addition of antioxidants commonly used in pharmaceutical products. The antioxidants used in this study were: methionine (95.2 μg/mL or 250 μg/mL), cysteine (95.2 μg/mL), glutathione (90.9 μg/mL), vitamin E TPGS (90.9 μg/mL), ascorbic acid (1.0 mg/mL), sodium ascorbate (1.0 mg/mL), and EDTA (1.0 μg/mL).

RC-101 concentration after exposure to preformulation conditions was analyzed by HPLC as previously described [11]. Briefly, the HPLC system (Waters Corporation, Milford, MA) was equipped with an autoinjector model 717, a quaternary pump model 600, and an ultraviolet (UV) detector model 2487 set up at 215 nm. Separation of RC-101 from degradant products was achieved using a Phenomenex Jupiter 5 μC5 300 Å (4.6 × 250 mm) column (Phenomenex, Torrance, CA) protected by a Widepore C5 (4 × 3.0 mm) guard cartridge (Phenomenex). The gradient consisted of mobile phase A (0.1% TFA in water (v/v)), and mobile phase B (0.07% TFA in acetonitrile (v/v)) pumped at a flow rate of 1.0 mL/min. Forced degraded samples (oxidation, temperature, and basic and acidic hydrolysis) were used to establish that the method could separate the degradants from the main peak.

Changes in the secondary structure of the protein were monitored by Circular dichroism (CD) on an AVIV Circular Dichroism spectrophotometer model 202 (AVIV Biomedical, Lakewood, NJ) equipped with a 0.1 cm path length quartz cell. RC-101 stability was also monitored by using a matrix-assisted laser desorption ionization-time of flight (MALDI-TOF) mass spectrometric (MS) on a Voyager DE-PRO mass spectrometer (Applied Biosystems, Foster City, CA). Potential for aggregation was evaluated by UV-spectroscopy using a NanoDrop ND-1000 spectrophotometer (NanoDrop Technologies Inc, Wilmington, DE).

### Human vaginal fluids collection protocol

Human vaginal fluid (HVF) was collected from 17 healthy premenopausal women according to protocol IRB number REN11050038/PRO07050142, approved by the Institutional Review Board under 45 CFR 46.110.(9). Inclusion criteria included ages between 18 and 45 years and agreeing to be abstinent from sexual activity for 48 hours prior to fluid collection. Women who were found to be pregnant, or to have used vaginal products or to have sexual intercourse in the 48 hours prior to collection were excluded. After signing informed consent and confirming eligibility, subjects completed a questionnaire and were then instructed on the use of the Instead Softcup^® ^(Instead Inc., La Jolla, CA) [12]. Instead Softcup^® ^is a FDA approved device to hold menstrual fluid during the menstrual period in replacement of a tampon or pad. Subjects inserted the cup and waited for 30 min. After this time period, the physician removed the cup, and placed it into a 50 mL conical centrifuge tube. Vaginal fluid collected from healthy volunteer women was stored at 4°C until used, and it was used within 4 h after collection. Usual volumes collected using the Softcup ranged from 0.1 to 0.8 mL depending on the subject.

After removing the Instead Softcup^®^, a speculum examination was performed. Swab specimens of the endocervix were obtained using the Mini-tip Culturette TN collection system (Becton Dickinson, Sparks, MD) according to the manufacturer's guidelines. *C. trachomatis *and *N. gonorrhoeae *were detected with an amplified DNA assay based on the simultaneous amplification and detection of target DNA amplification primers and a fluorescent label detector probe [13]. Bacterial vaginosis was detected by Gram stain and assessed by the Nugent score, where score results between 0 and 3 indicate a normal flora, between 4 and 6 indicates an intermediate state, and between 7 and 10 indicates bacterial vaginosis [14]. Subjects were notified of the test results by telephone within two weeks of collection and directed to the Allegheny County Public Health Department (Pittsburgh, PA) for treatment and additional testing, if needed.

### Preparation of RC-101 solution and film formulation

RC-101 100 μg/mL solutions were prepared by dissolving RC-101 in Milli Q water. RC-101 and placebo films were prepared by precasting a polymeric film solution into an 8-well-plate. The polymeric film solution was prepared as previously described [15] by adding Milli Q water, PVA, and hydroxypropyl methyl cellulose (HPMC). The solution was then heated at 95°C for 20 minutes for complete dissolution of the polymers. After cooling, glycerin and RC-101 were added. Film solution (2.4 g) containing RC-101 was poured into each well of the 8-well plate. The plate was placed into a vacuum oven at 30 ± 2°C for 20 ± 4 h. All dried films were removed from the plates and stored at room temperature in PET/Aluminum foil pouches (Amcor Flexibles Healthcare Inc, Mundelein, IL) until further analysis. Placebo films were prepared in the same way without the addition of RC-101. Each RC-101 film contained 100 μg of RC-101. For analytical purposes, films were dissolved in 1 mL of Milli Q water before addition to HVF.

### Preparation of RC-101 + HVF sample

The HVF from the Instead Softcup^® ^was removed by centrifugation of the conical tube for 10 min at 5,000 rpm. This first centrifugation allowed for an efficient removal of the HVF from the Instead Softcup^®^. HVF was then removed from the cup and the pH of each HVF sample was measured with Fisher Alkacid pH filter strips (Fisher Scientific). All samples collected on a specific day were pooled to be used for the research studies. If the pH of the individual samples was higher than 5, the sample was not included in the pool but it was stored at -80°C for separate analysis. If the sample contained blood, it was immediately discarded. Samples were prepared as described in Table 1. All samples (RC-101 solution, RC-101 film or placebo film) were combined with vehicle (HVF or water) in a ratio of 1:1. Because of its high viscosity, HVF was measured by weight and not by volume. All solutions were prepared fresh and incubated with HVF (or water) at 37°C for specific periods of time (0, 2, 6, 12, 24, 48, and 72 h), unless specified otherwise. At each time point, the samples were centrifuged at 10,000 rpm for 10 min to separate the supernatant from the epithelial cells as described in the cell processing section. Both parts (supernatant and cells) were stored at -80 ± C until analyzed by LC-MS/MS. To evaluate the influence of freezing the fluid prior to the analysis, the last pool of HVF was divided into two samples: one used fresh (at the time of collection), and the other one stored at -80°C for a 3-month period. After that time period, HVF was thawed and processed for blank and RC-101 solution only, as described in Table 1. HVF samples collected with a high pH value indicative of BV were stored at -80°C as previously mentioned. After confirmation of BV on those fluid samples by Gram stain score, the fluid samples (HVF BV^+^) were thawed, pooled, and processed as described in Table 1.

**Table 1:** Summary of RC-101, in solution and formulated, samples combined with HVF.

Sample Code	Sample Description	HVF	RC-101 (100 μg/mL) SOLUTION	Water	RC-101 100 μg/ FILM*	Placebo FILM*
A	Blank HVF	100 mg	-----	100 μL	-----	-----

B	RC-101 solution combined with HVF	100 mg	100 μL	-----	-----	-----

C	RC-101 solution control	-----	100 μL	100 μL	-----	-----

D	RC-101 film combined with HVF	100 mg	-----	-----	100 mg	-----

E	RC-101 film control	-----	-----	100 μL	100 mg	-----

F	Placebo film combined with HVF	Not performed	-----	-----	-----	-----

G	Placebo film control	-----	-----	100 μL	-----	100 mg

A_F_	Blank HVF frozen	100 mg	-----	100 μL	-----	-----

B_F_	RC-101 solution with frozen HVF	100 mg	100 μL	-----	-----	-----

A_BV+_	Blank HVF BV^+^	100 mg	-----	100 μL	-----	-----

B _BV+_	RC-101 solution with HVF BV^+^	100 mg	100 μL	-----	-----	-----

Amounts correspond to one time point.

*RC-101 film and placebo film were initially dissolved in 1 mL of water prior to addition to HVF. Amounts in the films columns correspond to the solution of the film in 1 mL of water.

### Sample processing for analysis

At each time point, the sample was removed from the incubation chamber and centrifuged for 10 min at 10,000 rpm, at 4 ± C to separate the supernatant from cell pellet. Supernatant (100 μL) was added to microcentrifuge filters Ultracel YM-10 Microcon MWCO 10,000 (Millipore Corporation, Bedford, MA), which were pre-washed with Milli Q water to eliminate any trace of propylene glycol from the filters. Samples were centrifuged twice for 15 min at 8,500 rpm, at 4 ± C. The filtrate was collected and frozen at -80 ± C until further analysis. A solution of 3 M urea was added to the cell pellet (1:1 w/w) obtained from the first centrifugation, to lyse the cells. This mixture was vortexed three times for 30 sec, and then centrifuged for 10 min at 10,000 rpm, at 4 ± C. The supernatant obtained from the cell lysate was then added to microcentrifuge filters Ultracel YM-10 Microcon MWCO 10,000 pre-washed with Milli Q water. Samples were centrifuged twice for 15 min at 8,500 rpm, at 4 ± C. The filtrate was collected and frozen at -80 ± C until analysis. The peptide RC-101 has been shown to be stable in 3 M urea for at least 24 h. Samples were thawed and added to PepCleanTM C-18 spin columns (Pierce Biotechnology Inc., Rockford, IL) for desalting, after column conditioning with acetonitrile:water (50:50) and equilibration with 0.1% trifluoroacetic acid. The column was washed three times with 0.1% trifluoroacetic acid, and RC-101 was eluted with acetonitrile:water (60:40) in 0.1% trifluoroacetic acid. Samples were dried in a speed vacuum CentriVap concentrator (LabConco Corp., Kansas City, MO) and resuspended with 200 μL of Milli Q water for LC-MS/MS analysis. Each sample described in Table 1 originated two sets of samples: one labeled as supernatant and the second one labeled as cells.

### Nanoflow Liquid Chromatography Selected Reaction Monitoring (SRM) Mass Spectrometry

Integrated electrospray ionization (ESI)-capillary reversed-phase columns (75 μm inner diameter × 360 μm outer diameter × 100 mm length) packed with 5 μm 300 Å pore size Jupiter C18 reversed-phase stationary phase (Phenomenex) were prepared, as previously described [16]. Solvent flow was supplied by a nanoflow HPLC system (Ultimate 3000, Dionex Corporation, Sunnyvale, CA). Each sample (3 μL) was loaded onto the column through a 5 μL loop at a flow rate of 0.5 μL/min in 98:2 mobile phase A (0.1% formic acid in water, v/v) and mobile phase B (0.1% formic acid in acetonitrile, v/v) for 30 min. The step-wise linear gradient was delivered at 250 nL/min as follows: 2 to 40% mobile phase B over 40 min, followed by 40 to 98% mobile phase B over 30 min. High voltage contact for ESI was provided through a metal union connecting the microcapillary column to the pump. The RC-101 peptide abundance was measured by SRM using a triple quadrupole MS (TSQ Quantum Ultra, Thermo Fisher Scientific Inc., San Jose, CA). While operating in SRM mode, Q1 and Q3 resolutions were set to 0.7 atomic mass unit (amu), and the collision induced dissociation (CID) gas pressure was 1.5 mTorr with a collision energy (CE) of 18 volts. Each SRM scan width was set to 0.002 m/z units and the scan rate was 0.020 sec. RC-101 peptide abundance was measured by selected reaction monitoring (SRM). Initially, confirmation of the peptide detection was obtained on a high resolution Orbitrap mass spectrometer (Thermo Scientific). The initial base peak chromatogram with a representative mass spectrum of the [M + 4H]^4+ ^RC-101 molecular ion was obtained (data not shown).

After the incubation period of RC-101 combined with HVF, each sample (described in Table 1) was removed from the incubation chamber and processed for LC-MS/MS analysis as described above. For each condition analyzed, supernatant and cells, the LC-MS/MS chromatogram was obtained. Data were analyzed by construction of mass chromatograms for each SRM transition separately, and peak areas were manually tabulated

### Statistical analysis

HPLC data obtained from the preformulation studies were expressed as the average percentage of the peak area from time 0 ± standard deviation, n = 3. Results were analyzed by one-way analysis of variance (ANOVA) with multiple comparisons of individual time points by using post hoc Bonferroni correction to detect significant differences under different conditions. P-values ≤ 0.05 were considered to be statistically significant, unless specified otherwise.

## Results and Discussion

Recently, several biopharmaceuticals (proteins and peptides) have been investigated as potential microbicides for prevention of HIV [6,17-19]. However, formulation and delivery of biopharmaceuticals can be difficult due to degradation and targeting challenges. A successful formulation will protect the peptide against degradation during the manufacturing process, during the shelf-life of the product, and after the protein enters the biological system [20,21]. According to the Alliance for Microbicide Development [2], several needs in microbicide formulation are considered to have a high priority, this includes preformulation evaluation. The current study addressed this issue by characterizing the stability of RC-101 and thereby informing the formulation development and, improving the efficacy of the product.

**Figure 1:**
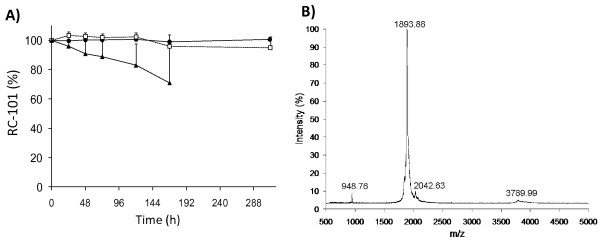
**Effects of temperature on RC-101 (500 μg/mL) solutions**. A) HPLC analysis for RC-101 stored at (solid circle) 25 ± C, (open square) 37 ± C, and (solid triangle) 65 ± C. B)MALDI-TOF MS spectrum of RC-101 in water, exposed for 10 days at room temperature, 100% intensity = 38291 counts.

RC-101 (MW = 1890.42) (GICRCICGKGICRCICGR) is a circular cationic 18-residue peptide, tetracyclic peptide with three cysteine disulfides bonds [22]. Preformulation studies showed that no statistically significance difference was observed for RC-101 stored at 25 and 37 ± C for a period of 13 days (p > 0.5), post hoc Bonferroni correction for multiple comparisons applied. Samples stored at 65 ± C showed a significant decrease in the amount of RC-101 at 168 h (p < 0.04) compared to RC-101 incubated for the same time period at 25 ± C (Figure 1A). MALDI-TOF MS was used to confirm the m/z of RC-101 (Figure 1B). Stability at 37 ± C suggests that the peptide will be stable at body temperature for a prolonged period of time. Protein stability at high temperatures should be considered not only to understand how the drug will be affected in the body, but also how the compound will behave during the manufacturing process when high temperature may be required for processing. In addition, this information would be useful to predict shelf-life. The data showing that RC-101 is susceptible to degradation at 65 ± C indicates that the manufacturing process of a RC-101 microbicide product should avoid prolonged exposure of the drug to high temperatures. However, chemical stability of RC-101 under temperature conditions is superior to several other proteins studied that showed fast thermal degradation at temperatures higher than 40°C [23,24].

**Figure 2:**
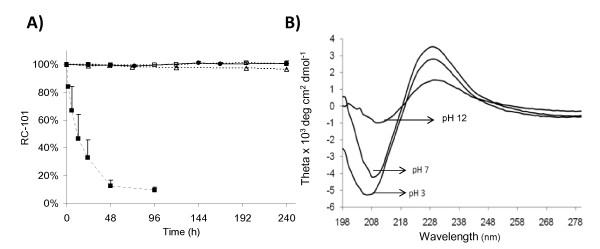
**Effect of pH on RC-101**. A) RC-101 under different pH conditions analyzed over time by HPLC. (open square) pH 3, (solid circle) pH 4, (open triangle) pH 7, and (solid square) pH 12. B) CD spectra of RC-101 solution (500 μg/mL) under different pH conditions.

The peptide RC-101 was shown to be stable in phosphate buffer solutions of pH 3, 4 and 7 using HPLC assay. Concentration of RC-101 by HPLC over time at different pH is shown in Figure 2A. Post hoc Bonferroni analysis for multiple comparisons was applied and no statistically significant decrease was observed over a period of 10 days for the samples at pH 3, 4, and 7 (p > 0.83). A significant decrease was observed at pH 12 in the first 2 h. CD was conducted on buffer solutions of 500 μg/mL RC-101 at pH 3, pH 7, and pH 12 (Figure 2B). Under all conditions, the protein showed a random conformation, with a maximum absorbance at 230 nm and a minimum absorbance at 200 nm for pH 3, 205 nm for pH 7, and 210 nm for pH 12. The peak shift in the wavelength and the loss of absorbance for pH 7 and pH 12 samples when compared to the pH 3 indicate a change in folding of the protein. However, the change observed at pH 7 did not affect bioactivity of RC-101 against HIV-1 (data not shown).

**Figure 3:**
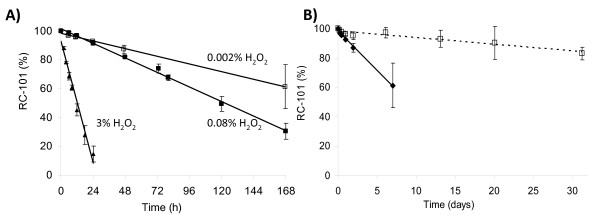
**Effect of hydrogen peroxide on RC-101**. A) RC-101 under different concentrations of hydrogen peroxide over time analyzed by HPLC. B) RC-101 exposed to hydrogen peroxide 0.002% without EDTA (solid circle), and in the presence of EDTA (open square), over time, analyzed by HPLC.

UV spectroscopy results for RC-101 with high ionic strength buffers (pH 4 and 7) did not show any significant differences in stability profiles, increasing the flexibility for formulation development. UV scans of RC-101 (500 μg/mL) in phosphate buffers pH 4, 7, and 12 were conducted (data not shown). Similar scans were observed for RC-101 pH 4 and 7; however, an increase in the absorbance at pH 12 samples was observed in the range of 300 to 600 nm, indicating the presence of aggregates. The stability of RC-101 in acidic pH is an important finding as the drug will be exposed to the acidic environment of the normal vagina with a pH (3.5 to 5.0). In addition, since the peptide is stable from pH 3 to 7, it expands the pH range for formulation of the microbicide product. This will be important for when the product is exposed to semen. The development of a successful peptide microbicide product is primarily dependent on the ability to prevent the oxidative effects of H_2_O_2_, present in the vaginal lumen. The stability of RC-101 was investigated under different levels of hydrogen peroxide. Forced degradation studies to evaluate oxidative effects are commonly conducted by exposing the molecule of interest to a solution of 3.0% H_2_O_2 _[25]. Results of RC-101 (500 μg/mL) exposed to 0.002, 0.08 and 3.0% hydrogen peroxide are shown in Figure 3A. RC-101 quickly degraded in the presence of 3.0% H_2_O_2 _(20% loss in 4 h). However, the degradation rate was slower in the presence of more biologically relevant concentrations (0.002% and 0.08% H_2_O_2_). Biologically relevant levels were selected based on reported studies which determined the amount of hydrogen peroxide produced by Lactobacillus present in the normal vaginal flora, and estimated calculations based on concentrations of Lactobacillus present [9,10]. RC-101 amino acid sequence contains six cysteines which are prone to oxidation; however the cysteines are present in their oxidized form, decreasing the likelihood of oxidative degradation. The intramolecular disulfide bonds may further oxidize resulting in sulfenic acid. The oxidation of the cysteine residues is a metal-ion catalyzed oxidation reaction. Most of the antioxidants used in this study did not show a significant protective effect against oxidation by the presence of hydrogen peroxide. Ethylenediamine tetraacetic acid (EDTA) was the only antioxidant investigated that showed protection of RC-101 against oxidation after exposure to H_2_O_2 _(Figure 3B). EDTA is a widely used chelating agent, approved by the Food and Drug Administration (FDA) as a preservative for pharmaceutical products.

Further formulation development may include the addition of EDTA. However, preliminary studies conducted in our laboratory have shown that EDTA is toxic to human ectocervical tissue and normal vaginal microflora in concentrations of 1% or higher (data not shown). Due to this fact, this preservative should be further characterized regarding its potential for toxicity *in vivo*.

Protection of RC-101 against oxidation may be necessary during the shelf-life of the final formulation and during the delivery in the vaginal lumen. The result from the addition of EDTA to the RC-101 solution is indicative of a method to protect RC-101 from oxidation during shelf-life of the product. In a biological environment, when the microbicide product is administered intra-vaginally, it will encounter the presence of vaginal fluids and cervical mucus that will not only dilute the microbicide agent, but also be a potential for degradation. The enzymatic activity present may initiate degradation of the peptide, in addition to the normal vaginal flora that produces hydrogen peroxide which will accelerate oxidation of RC-101. Our studies have shown that RC-101 is susceptible to oxidation, but in a very slow kinetic of degradation. Depending on the time for binding of RC-101 to receptors and glycoproteins, oxidation of RC-101 after 48 h may be an irrelevant degradation pathway and may not affect bioactivity. It is still unknown how long the drug should be active in the vaginal lumen, but it has been suggested that the virus stays in the vaginal lumen for a period of 48 h [26,27]. If that is the case, short-term protection of RC-101 may be sufficient to overcome oxidative degradation pathways in the vaginal lumen and guarantee biological activity.

An important factor is to investigate the stability of RC-101 in the presence of biological fluids. In this study, RC-101 was also investigated after combination with fresh undiluted human vaginal collected from healthy female volunteers.

Human vaginal fluid (HVF) was collected from a total of 17 female premenopausal women. The fluid collected represented individuals with a mean age of 31 ± 8 years. Average pH for normal fluid samples collected was 4.5 ± 0.6. None of the participants were using a vaginal ring or Intra Uterine Device (IUD) as contraceptive. None of the subjects tested positive for either *C. trachomatis *or *N. gonorrhoeae*. Samples from volunteers were pooled on the day of collection generating 3 pools (Pool 1, 2 and 3) for normal HVF, and one pool (BV pool) for HVF positive for BV. All the data obtained from the questionnaire was compiled for each pool and the most relevant data is presented in Table 2.

**Table 2:** Demographics of the subjects whose samples were pooled, per sample pool

Characteristic	Pool 1	Pool 2	Pool 3	BV Pool
Age				
Mean ± SD	28.7 ± 6.7	30.6 ± 8.9	33.2 ± 7.8	35.2 ± 5.2

pH	4.1 ± 0.3	4.3 ± 0.3	4.1 ± 0.4	5.8 ± 0.6

BV score				
Between 0 and 3	3 (75.0)	5 (71.4)	3 (50.0)	0
Between 4 and 6	1 (25.0)	2 (28.6)	1 (16.7)	1 (25.0)
Between 7 and 10	0	0	2 (33.3)	3 (75.0)

Last sexual intercourse				
Between 2 and 5 days prior	1 (25.0)	1 (14.2)	2 (33.3)	2 (50.0)
6 or more days prior	3 (75.0)	3 (42.8)	3 (50.0)	2 (50.0)
Not sexually active	0	3 (42.8)	1 (16.7)	0

Currently using vaginal products				
Yes (more than 2 days prior)	0	0	0	0
No	4 (100.0)	7 (100.0)	6 (100.0)	4 (100.0)

Several factors such as menstrual status, oral contraceptive use, and age will affect the amount and characteristics of vaginal fluids [28-30]. The questionnaire applied to all participant volunteers to characterize the demographics of the population included but was not limited to: day of the menstrual cycle, drinking status, and smoking status. Due to the number of volunteers used and the necessity to pool samples to obtain a significant volume for the analysis, we were unable to make any conclusions regarding the demographics information collected and the stability of RC-101 in the fluids.

**Figure 4:**
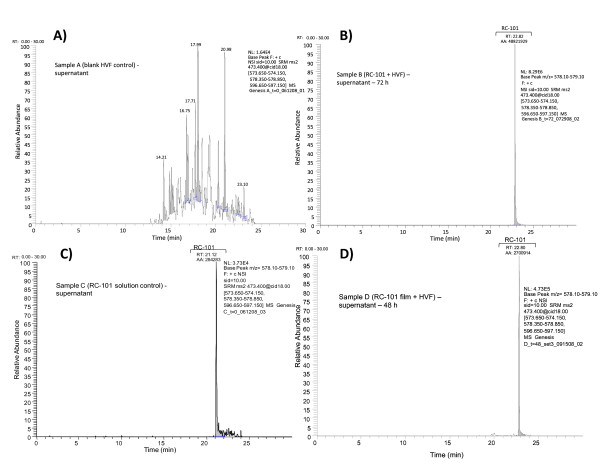
**Representative LC-MS/MS chromatograms for A) Sample A supernatant (blank HVF) at time 0, B) Sample B supernatant (RC-101 solution + HVF) at 72 h, C) Sample C supernatant (RC-101 solution control), and D) Sample D (RC-101 film + HVF) supernatant at 48 h**.

This is the first study in the microbicide field to evaluate a microbicide candidate using fresh HVF. After the incubation of RC-101 with HVF, abundance of the peptide was measure by LC-MS/MS. Representative LC-MS/MS chromatograms at time 0 are shown in Figure 4 for Sample A supernatant (blank HVF), Sample B supernatant (RC-101 solution + HVF) at 72 h, Sample C supernatant (RC-101 solution control), and Sample D (RC-101 film + HVF) at 48 h. Sample A (HVF control) showed the presence of several peaks; however, no interference peaks were detected, indicating that the method was suitable for detection of RC-101. For all other chromatograms, the m/z was confirmed for RC-101 detection. Since the LC-MS/MS method developed is not a quantitative method, the amount of RC-101 was not obtained. Overall, RC-101 was detected for 48 h in two pools tested and up to 72 h in another pool tested. Formulation of RC-101 into the film still maintained the stability of RC-101 over the same time period. Overall, RC-101 was detected after exposure to HVF at least for 48 h, and no difference was observed for RC-101 in a solution or a film formulation.

To verify if the freezing process would interfere with the stability of RC-101 in the fluid, frozen HVF was used for incubation with RC-101 solution (Sample BF). It was expected that RC-101 would be detected at a higher concentration when using frozen HVF, due to the suspected decrease in enzymatic activity of the fluid upon freezing. Since the LC-MS/MS method is not quantitative, it was not possible to determine this difference in concentration. No detectable differences were observed in the peptide after incubation with frozen fluid.

Stability of RC-101 over time was also investigated in bacterial vaginosis (BV) fluid obtained from volunteers (HVF BV^+^). These samples were collected and stored at -80°C, prior to incubation with RC-101. When RC-101 was combined with HVF BV^+ ^Pool (samples BBV^+^), RC-101 was undetectable in the LC-MS/MS analysis at any time point studied, demonstrating that RC-101 was not stable in those fluids. No RC-101 was detected at any time point in the Sample BBV^+ ^in either supernatant or cells. The results are summarized in Table 3.

**Table 3:** Summarized results for detection of RC-101 by LC-MS/MS

		Time (h)						
**Sample Code**	**Sample Description**	**0**	**2**	**6**	**12**	**24**	**48**	72*

A	Blank HVF	**-**	**-**	**-**	**-**	**-**	**-**	**-**

B	RC-101 solution combined with HVF	**+**	**+**	**+**	**+**	**+**	**+**	**+**

C	RC-101 solution control	**+**	**+**	**+**	**+**	**+**	**+**	**+**

D	RC-101 film combined with HVF	**+**	**+**	**+**	**+**	**+**	**+**	**+**

E	RC-101 film control	**+**	**+**	**+**	**+**	**+**	**+**	**+**

G	Placebo film control	**-**	**-**	**-**	**-**	**-**	**-**	**-**

A_F_	Blank HVF frozen	**-**	**-**	**-**	**-**	**-**	**-**	NA

B_F_	RC-101 solution with frozen HVF	**+**	**+**	**+**	**+**	**+**	**+**	NA

A_BV+_	Blank HVF BV^+^	**-**	**-**	**-**	**-**	**-**	**-**	NA

B _BV+_	RC-101 solution with HVF BV^+^	**-**	**-**	**-**	**-**	**-**	**-**	NA

(-) represents absence of RC-101 peak; (+) represents presence of RC-101 peak detected by LC-MS/MS. *only one pool of sample analyzed up to 72 h. NA = not analyzed samples at this time point.

If RC-101 can be detected in HVF for at least 48 h, it is suggested that RC-101 will be available for binding to gp120 during that time period, conferring protection against HIV. The prolonged stability of RC-101 in HVF indicates that this molecule is a promising candidate to be delivered vaginally and can survive the enzymatic activity present in normal vaginal fluid. However, further studies *in vivo *are recommended to confirm the results obtained. Another advantage of the stability of RC-101 for at least 48 h in HVF is the dose regimen selected for the microbicide. The stability suggests that the final RC-101 microbicide product could be applied once every two days or once a day, without being coitally-dependent. This would increase patient adherence to the product, which may be more favorable to a successful product. As a future study, the RC-101 detected after incubation with HVF should be tested for bioactivity against HIV.

The impact of HVF positive for bacterial vaginosis (BV) has also been investigated. It has been shown that RC-101 was completely unstable in fluid positive for BV evidenced by the undetectable levels of RC-101 after exposure to HVF positive for BV at all time points. Some studies have evaluated the difference between normal HVF and HVF positive for BV, and a difference in the enzymatic activity between a normal fluid and a BV positive fluid has been demonstrated [9,30-32]. BV is characterized by a reduction in vaginal colonization by Lactobacillus and an overgrowth of anaerobic gram-negative bacteria. Intensive production of hydrolytic enzymes in BV [31-33] may lead to a decreased mucosal barrier in the vaginal and cervical mucosa. The higher enzymatic activity found in BV might explain the immediate degradation of RC-101 in the presence of HVF positive for BV. In addition, electrostatic interactions between cationic peptides and the anionic surface of bacteria may occur [34], leading to possible adherence of RC-101 to the BV bacteria which may explain the decrease in the presence of RC-101. This finding is extremely important for designing future studies for the development of biopharmaceuticals and other molecules as microbicides. Bacterial vaginosis is a highly prevalent condition, affecting almost one third of women between the ages of 14 and 49 years old in the United States, according to the 2001 - 2004 National Health and Nutrition Examination Survey [35]. Considering the high prevalence of BV, further studies should investigate the effects of HVF positive for BV on the stability of microbicide drug candidates. Furthermore, more advanced drug delivery strategies focused on protection of RC-101 from BV positive fluids, such as encapsulation of RC-101 in nanoparticles, may be needed prior to consideration of application in this population of women.

Another point to be considered is the rectal use of microbicide. Although rectal delivery was not part of the scope of our research, we understand that microbicide formulation development should consider the stability of the active microbicide ingredient in the presence of rectal fluids.

## Conclusions

This study has characterized the degradation pathways of RC-101 under various conditions, which are essential for the development of an effective microbicide product. It was shown that the microbicide drug candidate RC-101 is stable over a wide range of pH, temperatures and concentrations of hydrogen peroxide. RC-101 remained present in human vaginal fluid (HVF) for at least 48 h after incubation at 37°C, suggesting that RC-101 would be stable in this biological fluid. Formulation of RC-101 into a film maintained the stability of RC-101 in HVF for the same time period. However, it was found that the presence of BV in HVF considerably affects the stability of RC-101. Given the favourable results from the preformulation studies showing RC-101 to have a favourable stability profile and potential for achieving long term drug presence in the biological compartment RC-101 has great potential to advance in development as a microbicide drug candidate. Furthermore, the results described in this study underscore the importance of assessing the impact of human vaginal fluid on all potential microbicide products during the development process.

## List of abbreviations

BV: bacterial vaginosis; CD: circular dichroism; HPLC: high performance liquid chromatography; HPMC: hydroxypropyl methyl cellulose; HVF: human vaginal fluid; HVF BV^+^: human vaginal fluid positive for bacterial vaginosis; MALDI-TOF MS: matrix-assisted laser desorption/ionization - time-of-flight mass spectrometry; PVA: Polyvinyl alcohol; STIs: sexually transmitted infections.

## Competing interests

The authors declare that they have no competing interests.

## Authors' contributions

ABS has designed the experimental study and drafted the fluid collection protocol, collected human samples, carried out the majority of the experiments, and drafted the manuscript. KEB participated in writing the fluid collection protocol and has made substantial contribution in performing the human samples collection. BLH and TPC have made substantial contribution in developing and conducting the analysis for the LC-MS/MS method for protein detection in biological fluids. AMC and PG have participated in the conception and design of the study, and data interpretation. LC has made significant contributions to the overall concept of the study, experimental design, data interpretation, and final revision of the manuscript. All authors read and approved the final manuscript.
